# Maternal monosaccharide diets evoke cognitive, locomotor, and emotional disturbances in adolescent and young adult offspring rats

**DOI:** 10.3389/fnut.2023.1176213

**Published:** 2023-05-09

**Authors:** Kacper Witek, Karolina Wydra, Agata Suder, Małgorzata Filip

**Affiliations:** Department of Drug Addiction Pharmacology, Maj Institute of Pharmacology Polish Academy of Sciences, Kraków, Poland

**Keywords:** anxiety, behavior disorders, fructose, glucose, cognition, depression, hyperactivity

## Abstract

Anxiety and depression are the most common mental disorders affecting people worldwide. Recent studies have highlighted that a maternal high-sugar diet (HSD) could be a risk factor for neurobehavioural dysregulations, including mood disorders. Increased consumption of added sugar in food such as refined fructose/glucose can increase the risk of metabolic disorders and impact susceptibility to mental disorders. Furthermore, a few papers have reported disabilities in learning and memory among offspring after maternal HSD, thus suggesting a relationship between maternal nutrition and offspring neurogenesis. In this study, we evaluated the impact of maternal monosaccharide consumption based on a glucose (GLU) or fructose (FRU) diet during pregnancy and lactation in adolescent and young adult offspring rats of both sexes on cognitive, locomotor, and emotional disturbances. Locomotor activity, short-term memory, anxiety-like and depressive-like behavior were evaluated in the offspring. We report for the first time that the maternal GLU or FRU diet is sufficient to evoke anxiety-like behavior among adolescent and young adult offspring. Moreover, we found that maternal monosaccharide diets lead to hyperactivity and depressive-like behavior in male adolescent rats. We also noticed that a maternal FRU diet significantly enhanced novelty-seeking behavior only in young adult male rats. Our novel findings indicated that the maternal monosaccharide diet, especially a diet enriched in FRU, resulted in strong behavioral alterations in offspring rats at early life stages. This study also revealed that male rats were more susceptible to hyperactivity and anxiety- and depressive-like phenotypes than female rats. These results suggest that maternal monosaccharide consumption during pregnancy and lactation is an important factor affecting the emotional status of offspring.

## 1. Introduction

Mental disorders are diseases characterized by a clinically significant disturbance in an individual's cognition, emotional regulation, or behavior that reflects a dysfunction in the psychological, biological, or brain developmental processes (1). Mental disorders are global problems in modern society, and they affect people across genders and ages, imposing significant barriers to a person's ability to function normally. In 2019, one in every eight people, or 970 million people worldwide, lived with a mental disorder, among which anxiety and depressive disorders were the most common (2). On the other hand, it was estimated that in the United States, young adults aged 18–25 years had a higher prevalence of serious mental illness than adults aged 26–49 years and older (3). The development of mental disorders is due to biological, psychological, and social factors, and these risk factors may manifest differently at different ages and stages in life, with some of these risks appearing as early as the prenatal period (4). The Developmental Origins of Health and Disease hypothesis indicates that fetal exposure during a critical period of development and growth to external (environmental) and internal (maternal) factors can significantly impact the short- and long-term health of the offspring (5). Maternal diet during pregnancy and lactation can lead to metabolic or neurological diseases among offspring (6, 7).

Foods that contain sugar such as glucose (GLU) and fructose (FRU) are an important source of energy for cells and crucial for the maintenance of the body's biochemical processes. The brain consumes ~ 20% of GLU-derived energy, making it the main consumer of GLU (8). Both GLU and FRU act as sweeteners and added sugars in many food products, such as FRU in high-fructose corn syrup (HFCS) or GLU in sugar-sweetened beverages (SSB). Importantly, youth and adults consume calories from added sugar in processed food (9, 10). Moreover, many adults exceed the daily intake of free sugars, which is limited by the World Health Organization to < 10% of one's total energy intake (11). More than 10% of Americans' daily calories come from FRU consumption, which has potentially more harmful effects on the body than other major caloric sweeteners (12). Clinical observations have indicated that sugar-rich foods are desirable during pregnancy and were consumed too often during the study period, suggesting that many pregnant women were exposed to the adverse effects of a high-sugar diet (HSD) (13).

In a recent review, perinatal and postnatal HSD (such as sugar, sucrose, fructose, or glucose) consumption was considered a potential risk factor for neurobehavioural dysregulations such as mental disorders (14). Although several preclinical studies have evaluated the effects of maternal HSD on offspring behavior (15–18), most of these studies were performed using male or female rodents independently. It is also worth adding that most preclinical studies have focused on the relationship between postnatal HSD consumption and behavioral disturbances (19–24). However, maternal diet in the prenatal period also plays a crucial role in offspring neurodevelopmental disorders (25–27). Human observational studies have shown that stress-induced sugar or SSB consumption was correlated with increased anxiety and depression in adolescents and adults (28–31). Furthermore, HSD and sugar itself can be addictive and can predispose an individual to the risk of food addiction (32). Finally, sugar intake includes numerous neural mechanisms within the mesocorticolimbic system, hypothalamic orexigenic and anorexigenic pathways, and hypothalamic–pituitary–adrenal axis, all of which are strongly correlated with mental disorder development (33).

An increase in sugar consumption and several cognitive, locomotor, and emotional disturbances after maternal HSD suggest an interaction between these variables. However, there are no studies that have reported the effect of particular monosaccharides in the maternal diet on behavioral phenotypic changes in both sexes of offspring at later stages of life. Similarly, there are few preclinical studies have specifically investigated the impact of maternal HSD intake on offspring cognition and anxiety-like or depressive-like behavior development, while the impact of a maternal high-fat diet has been widely studied in this context. Hence, this study aimed to examine the effect of a maternal GLU or FRU diet during pregnancy and lactation on behavioral outcomes in adolescent and young adult unrelated offspring rats of both sexes.

## 2. Materials and methods

### 2.1. Experimental animal and maternal diets

Wistar rats obtained from the licensed animal Charles River breeder (Sulzfeld, Germany) were housed in standard plastic rodent cages in an animal breeding room at 21 ± 2°C and 40 ± 10% humidity with a 12-h light-dark (LD 12:12) cycle (lights on at 6:00 a.m.). Animals had free access to water and food *ad libitum*. Twenty-four virgin female rats (201–225 g), after the acclimatization period (14 days) and during the proestrus phase (the oestrous cycle phases determined by daily vaginal smears), were mated with six randomly assigned males (226–250 g) until the presence of sperm in the smears was confirmed. Pregnant females were individually housed and randomly assigned to one of three different experimental condition groups: standard control/VRF1 diet (SD), modified glucose (GLU) diet, or modified fructose (FRU) diet (Table 1). All diets were delivered by Special Diets Services (SDS; London, UK).

**Table 1:** Composition of main macronutrients (shown as a percentage of energy) and energy value of maternal experimental diets during pregnancy and lactation periods.

**Diet**		**Control/VRF1 (SD)**	**Glucose (GLU)**	**Fructose (FRU)**
	Pectin (%)	1.37	0.00	0.00
	Hemicellulose (%)	8.76	0.13	0.13
Carbohydrates	Cellulose (%)	3.95	6.40	6.21
		Lignin (%)	1.06	0.00	0.00
	Starch (%)	35.41	0.00	0.00
	Sugar (%)	4.64 (from sucrose)	53.76 (from glucose)	54.35 (from fructose)
Protein (%)		19.11	19.13	18.56
Fat (%)		4.75	5.06	4.91
Total energy (kcal/g)		3.40	3.44	3.43

The glucose (GLU) and fructose (FRU) diets are synthetic mimics of the control (SD) diet. The type of sugar was carefully modified and formulated to match the VRF1 diet.

**Figure 1:**
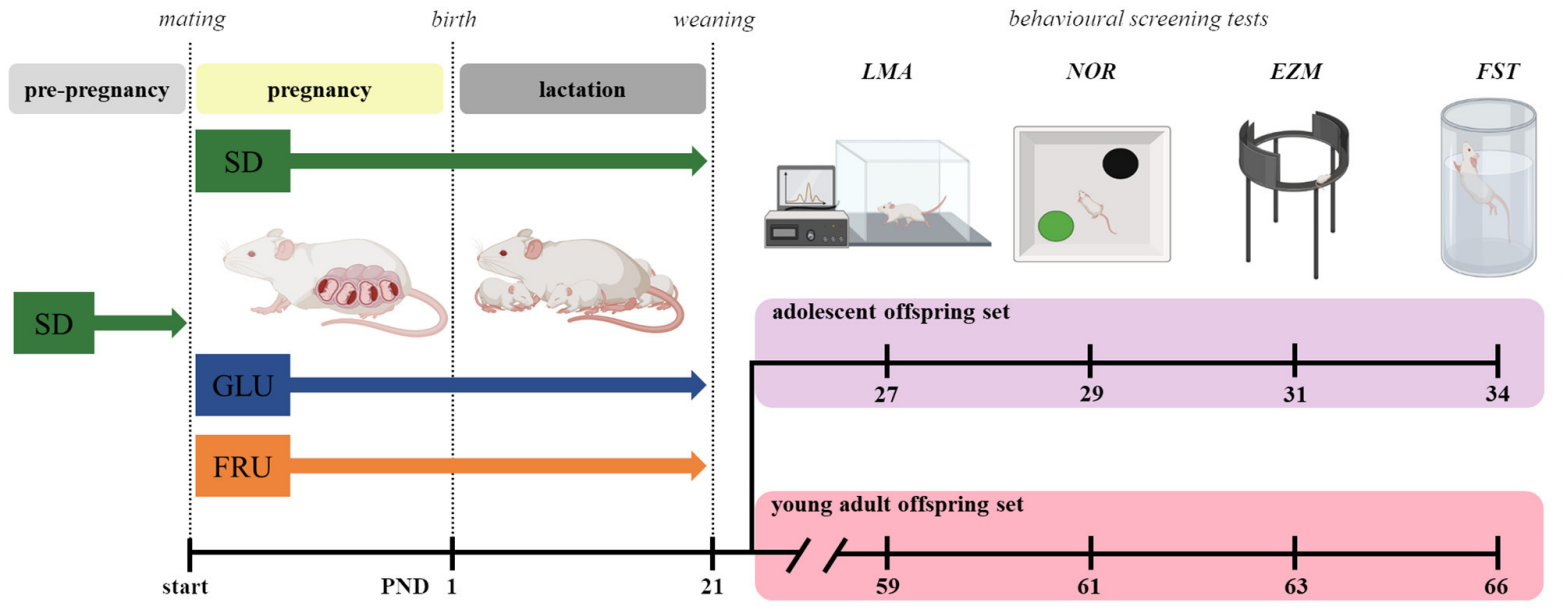
Scheme of the experiment. Dams before mating (pre-pregnancy) were fed a standard diet (SD) or, during pregnancy (~21 days) and lactation (~21 days), one of three diets: control (SD), glucose (GLU), or fructose (FRU). After weaning at postnatal day (PND) 21, the offspring were divided into two sets: adolescent and young adult. From each set, one offspring subset was used in behavioral screening tests: locomotor activity (LMA), novel object recognition (NOR), elevated zero maze (EZM), and forced swimming test (FST). All pups were fed SD chow after weaning.

During pregnancy (21 days) and lactation (21 days), dams were given *ad libitum* access to water and food, depending on the diet group (Figure 1). One day after birth, the pups were weighed, and the litter sizes were counted and normalized to 8–10 pups. Next, at postnatal day (PND), 21 offspring were weaned and separated according to sex, marked, housed 5 per cage, and switched to SD. At the same time, we took two offspring sets (selected from 7 to 8 different dams and six different males) to minimize the parental genetic influence and reduce individual offspring predisposition by increasing their variability in cohorts (34). For behavioral studies, we used one of two subsets of each set randomly consisting of eight male and eight female rats for each diet. Two independent offspring subsets were used to assess spontaneous behavior in the early life stage periods without exposure to earlier experimental conditions. Female and male rats were tested separately. All experiments were carried out in conformity with the European Union Directive (2010/63/EU), Polish Act on the Protection of Animals (Dz.U. z 2020 r. poz. 638), and with approval of the Local Ethics Commission (Kraków, Poland; approval numbers 18/2021 and 54/2021) with the three Rs rule.

### 2.2. Offspring behavioral tests

#### 2.2.1. Locomotor activity

Locomotor activity (LMA) was recorded for each non-habituated rat using the procedure described previously by Wydra et al. (35). Briefly, rats at PND 27 (subset from set I) and PND 59 (subset from set II) were placed individually in Opto-Varimex (43 × 43 cm) cages (Columbus Instruments, Columbus, USA). Spontaneous locomotor activity, measured as horizontal activity, was recorded for 2 h using the Auto-Track system (Columbus Instruments, USA) every 5-min trial and presented as the distance traveled (cm).

#### 2.2.2. Novel object recognition

To evaluate the ability to recognize a novel object (NOR) in the environment and working short-term memory in rats at PND 29 (subset from set I) and PND 61 (subset from set II), the novel object recognition test described previously by Gawlińska et al. (36) was used. On the first day, after 1 h of acclimatization in the experimental room, rats were placed separately for 10 min in the center of the empty, non-transparent plastic box (57 × 37 × 30 cm) arena for habituation. Next, for 5 min, the animals were presented with two identical objects—A, as a familiarization phase [A + A]. After a 1 h break in the home cages, rats again were placed in the same plastic box for 5 min and presented two objects, the familiar (F)—A and the first novel (N1)—B object, as a recognition phase I [A + B]. On the second day, 24 h after the familiarization phase, the rats were placed in the arena for 10 min and again presented with two objects: the previous (A) and the second novel (N2) (C) object as a recognition phase II [A + C]. Active object exploration was defined as time spent sniffing, touching, and close approach, not including the time spent sitting on the objects. The percentage of time spent exploring the novel object relative to the total time spent exploring both objects was measured using the recognition index (RI) formula: RI = TN/(TN + TF) × 100 (%), where T was the time spent exploring the novel (N1 or N2) and familiar (F) objects. An RI above 50% indicated novel object preference, below 50% indicated familiar object preference and 50% indicated no preference (37, 38). The following objects were presented during the test: black (F) and orange (N1) metal cans (13.5 cm high, 5 cm in diameter) and a violet glass block (6 cm high, 3–5 cm in diameter) with a green plastic cone (N2; 2 cm high, 3–5 cm in diameter). Objects were placed on two opposite sides of the box, 13 cm from the center of the arena and 22 cm from each object. Plastic boxes and objects were cleaned using 20% ethanol to minimize olfactory cues before the next round. To reduce the stress of a bright lighting room, areas were illuminated with 170–230 lux light (1 m above the box). The test was video recorded by one investigator, while the results were manually extracted from videos by two independent blinded assessors.

#### 2.2.3. Elevated zero maze

Anxiety-like behavior in rats was assessed using the elevated zero maze test (EZM) (39). The annular, black platform maze (105 cm in diameter, 10 cm path wide) consisted of four quadrants: two opposite open (with 1 cm high walls) arms and two opposite closed (with 25 cm high walls) arms, which were elevated to 65 cm above the floor. The rats were placed individually, alternately in one of the two closed arms of the maze, and a 5-min test was recorded. Behavioral measures comprised the time (s) spent in and the number of entries into open areas, the frequency of head dips from the dark to the light zone, beyond the wall in the open zone, and the frequency of stretch postures from closed to open quadrants. The maze was illuminated by a light source (120–150 lux) suspended 50 cm from the maze path. The test was conducted in the experimental, dark room after 1 h of acclimatization, 5 min of habituation to the surface, and color of the maze in the EZM box for PND 31 (subset from set I) and PND 63 (subset from set II) rats. The maze was cleaned using 20% ethanol before the next trial. All tests were performed and manually assessed by two independent investigators who remained at an appropriate distance of 2 m from the maze in the experimental room.

#### 2.2.4. Forced swim test

The modified forced swim test (FST) was used to assess offspring rats' responses to stress at PND 34 (subset from set I) and PND 66 (subset from II) (40, 41). On the first day (pre-test), after 1 h of acclimatization, adolescent rats were placed individually in glass-transparent cylinders (41 cm high, 20 cm diameter) containing clean water (25 ± 1°C) at a depth of 30 cm (preventing touching the bottom) for 15 min. Then, all rats were removed from the water, dried, warmed, and returned to their home cages. Twenty-four hours after the pre-test, the rats were retested for 5 min under the same conditions, where one investigator recorded each animal's activity using a digital camera. Each cylinder was emptied and cleaned before the test for the next rat. Next, two independent blinded assessors manually extracted from the video the time spent swimming, climbing, and immobility. Immobility time was observed when the rat floated in water without struggling and made only the movements necessary to keep its head above water. Swimming time was defined as active swimming motions (displaced body around the cylinder), more than necessary to merely maintain head above water. Climbing time was measured when the rat was climbing, making active movements with forepaws in and out of the water.

### 2.4. Statistical analysis

All experimental data are expressed as the mean ± SEM (standard error of the mean). Statistical analyses were performed with Statistica 13.3 (TIBCO Software Inc., Palo Alto, CA, USA) and visualized in GraphPad Prism 9.4.1 (GraphPad Software, Inc., San Diego, CA, USA). The Shapiro–Wilk test and Levene test were used to test the normality and equal homogeneity of variance, respectively. For normally distributed data, two-way analyses of variance (ANOVA: diet + gender + diet × gender; *F* statistic) were performed. Alternative two-way ANOVA with Welch's correction (*W* statistic) was used for unequal variances. For non-normally distributed data, a non-parametric Kruskal–Wallis test by ranks (*H* statistic) was performed. In significant differences between diet (compared to SD), either parametric Dunnett's and Dunnett's T3 or non-parametric Dunn's multiple comparisons *post hoc* tests were performed. For the sex-specific changes, parametric Šidák's or Dunnett's T3 multiple comparisons *post hoc* tests were performed. “*N*” corresponds to the number of individuals. *p* < 0.05 was considered statistically significant.

## 3. Results

### 3.1. Effect of monosaccharide diets on body weight and caloric intake

**Figure 2:**
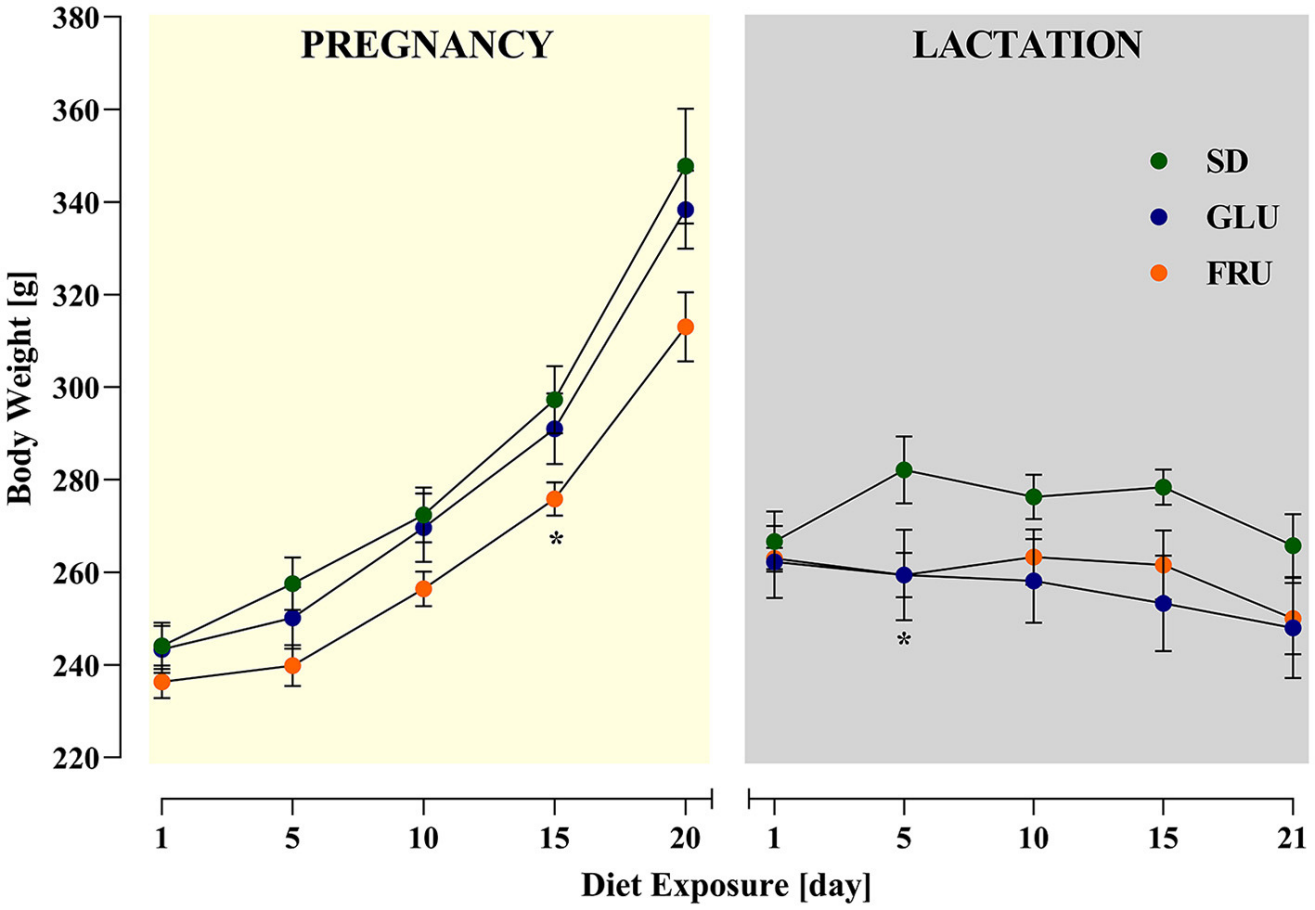
Effects of maternal glucose (GLU) or fructose (FRU) diet intake on dam body weight during pregnancy and lactation. At 15 days of pregnancy and 5 days of lactation, FRU dams showed less body weight than SD dams. *N* = 8 dams/SD and GLU, 7 dams/FRU. Data are comparable to the SD groups (*p < 0.05, two-way repeated measure ANOVA followed by Dunnett's multiple comparison test).

**Figure 3:**
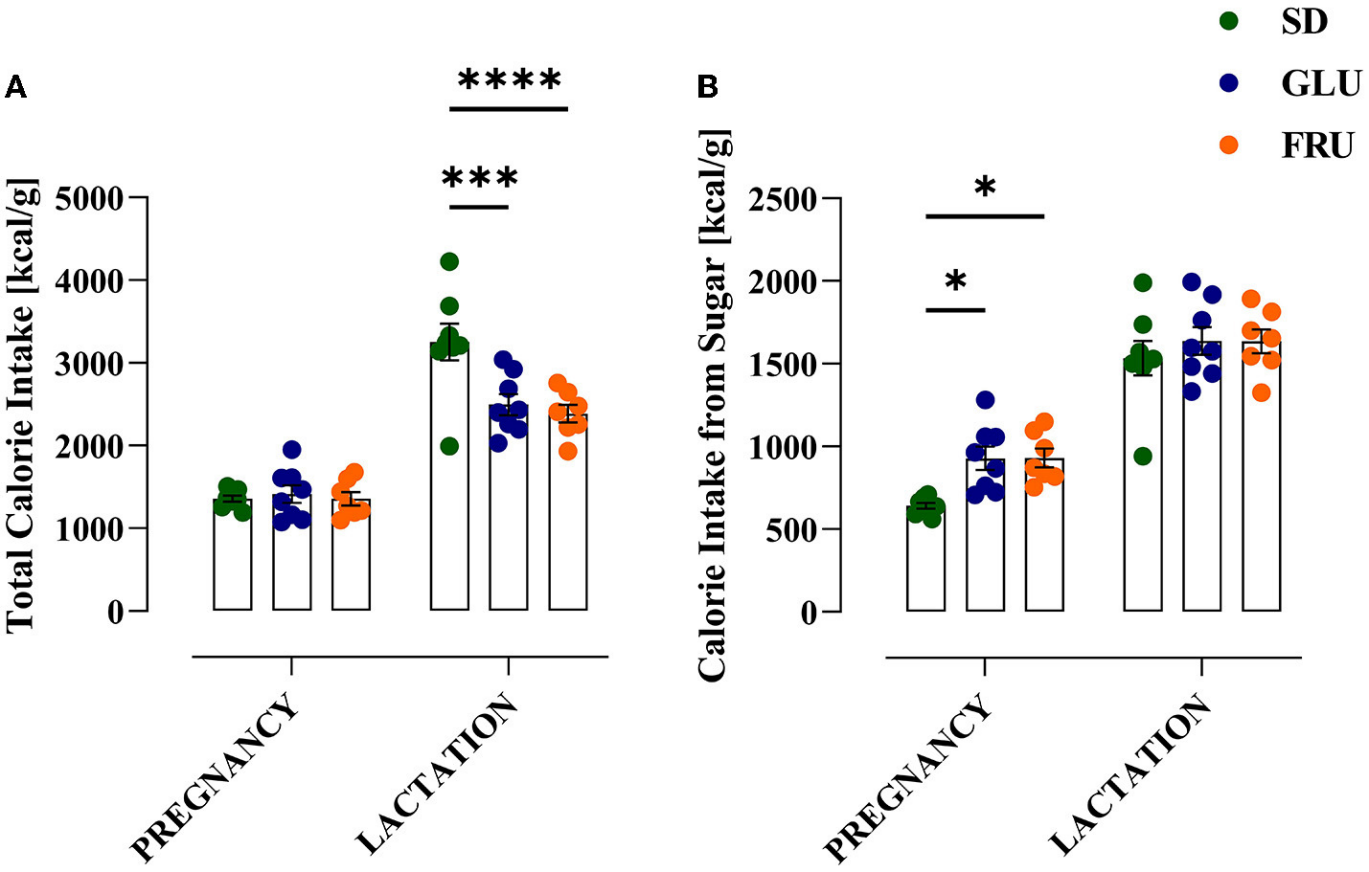
Effects of maternal glucose (GLU) or fructose (FRU) diet intake on dam calorie intake. **(A)** Total calorie intake during pregnancy and lactation. Both GLU and FRU dams had reduced total calorie intake during lactation but not during pregnancy compared to SD dams. **(B)** Calorie intake from a sugar source. Both GLU and FRU dams consumed more calories from sugar during pregnancy but not during lactation compared to SD dams. *N* = 8 dams/SD and GLU, 7 dams/FRU. Data are comparable to the SD groups (*p < 0.05, ***p < 0.001, ****p < 0.0001, two-way repeated measure ANOVA followed by Dunnett's multiple comparison test).

**Figure 4:**
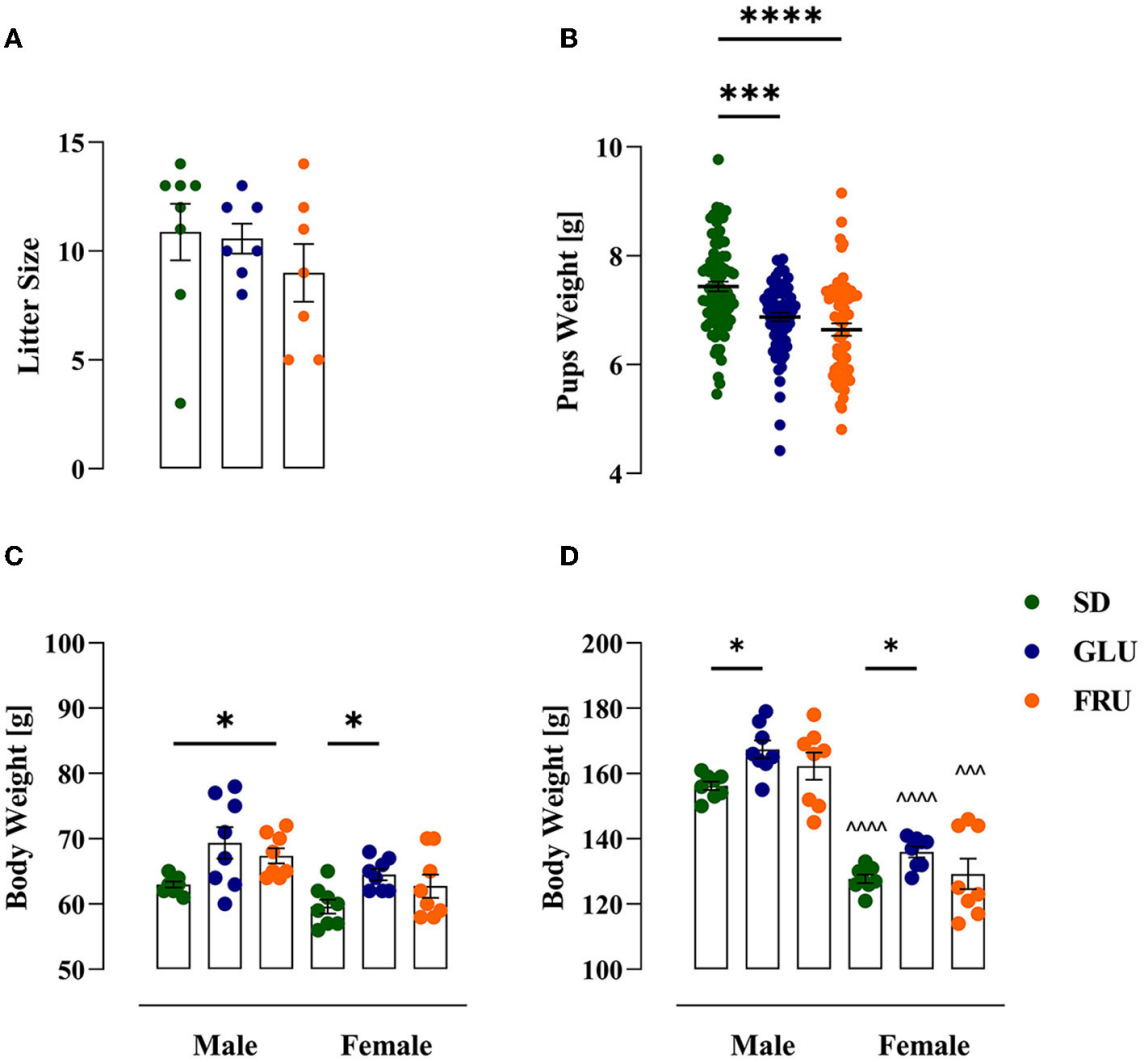
Effects of maternal glucose (GLU) or fructose (FRU) diet on litter size and offspring weight. **(A)** Litter size of each dam. Maternal diet affects litter size individually, but in total, no significant difference was observed. *N* = 8 dams/SD, 7 dams/GLU and FRU (Kruskal–Wallis tests). **(B)** Pups weight one day after birth (at PND2). GLU and FRU offspring rats weighed less than SD pups. *N* = 87 pups/SD, 74 pups/GLU and 63 pups/FRU. Data are comparable to the SD groups (^***^p < 0.001, ^****^p < 0.0001, Kruskal–Wallis tests followed by Dunn's multiple comparison test). **(C)** Body weight of adolescent (at PND27) offspring. FRU male and GLU female adolescents had higher body weights than SD offspring. **(D)** Body weight of a young adult (at PND59) offspring. Both GLU male and female young adults had higher body weights than SD offspring. Young adult females had reduced body weight compared to males. *N* = 8 rats/group. Data are comparable to the SD groups (*p < 0.05, two-way ANOVA with Welch's correction followed by Dunnett's T3 multiple comparison test) or between sexes in the same group (^∧∧∧^*p* < 0.001, ^∧∧∧∧^*p* < 0.0001, two-way ANOVA with Welch's correction followed by Dunnett's T3 multiple comparison test).

During the experiment, dams showed a significant main effect of the period [*F*_(1.634, 32.67)_= 106.90; *p* < 0.0001] and interaction [*F*_(18,180)_= 2.346; *p* = 0.0024] during 5-day measurements of body weight (Figure 2). During pregnancy (on day 15) and lactation (on day 5), SD dams had 8% and 9% higher body weights, respectively, than FRU dams (Figure 2). Moreover, we observed a main effect of the period [*F*_(1,40)_= 159.50; *p* < 0.001], diet [*F*_(2,40)_= 6.44; *p* = 0.0038] and interaction [*F*_(2,40)_= 7.13; *p* = 0.0023] on total calorie intake (Figure 3A). The GLU and FRU dams showed 23 and 27%, respectively, less total calorie intake than SD dams in the lactation period (Figure 3A). The total calorie intake was similar in the pregnancy period between the groups. Assuming that 1 g of sugar has 4 kcal, and the percentage of sugar in the diet has been described (Table 1), we also assessed the contribution of calories from the sugar in the total calorie intake (Figure 3B). Here, period [*F*_(1,40)_= 164.70; *p* < 0.001] and diet [*F*_(1,40)_= 4.87; *p* = 0.0128] effects were observed. Conversely, for total caloric intake, both GLU and FRU dams showed 31% higher calorie intake than SD dams in the pregnancy period (Figure 3B). No changes were observed in the lactation period. Although we observed different litter sizes between dams in each diet, these changes were not statistically significant [*H*_(2,N = 23)_ = 1.32, *p* = 0.5146; Figure 4A]. In turn, we observed significant differences in pup weight [*H*_(2,N = 224)_ = 30.69, *p* < 0.0001; Figure 4B] between diet groups. The GLU and FRU pups weighed less by 7 and 11%, respectively than the SD offspring (Figure 4B). Moreover, adolescent FRU male and GLU female offspring displayed 7 and 8%, respectively, higher body weights than SD rats [*W*_(5,18.66)_ = 5.73, *p* = 0.0023; Figure 4C]. Both GLU male and female young adult offspring had 6% higher body weight than SD offspring. Additionally, SD, GLU, and FRU female rats demonstrated 18%, 19%, and 20% lower body weight, respectively, than male rats in the same diet group [*W*_(5,19.14)_ = 64.52, *p* < 0.0001; Figure 4D].

### 3.2. Effects of maternal HMD on offspring locomotor activity

**Figure 5:**
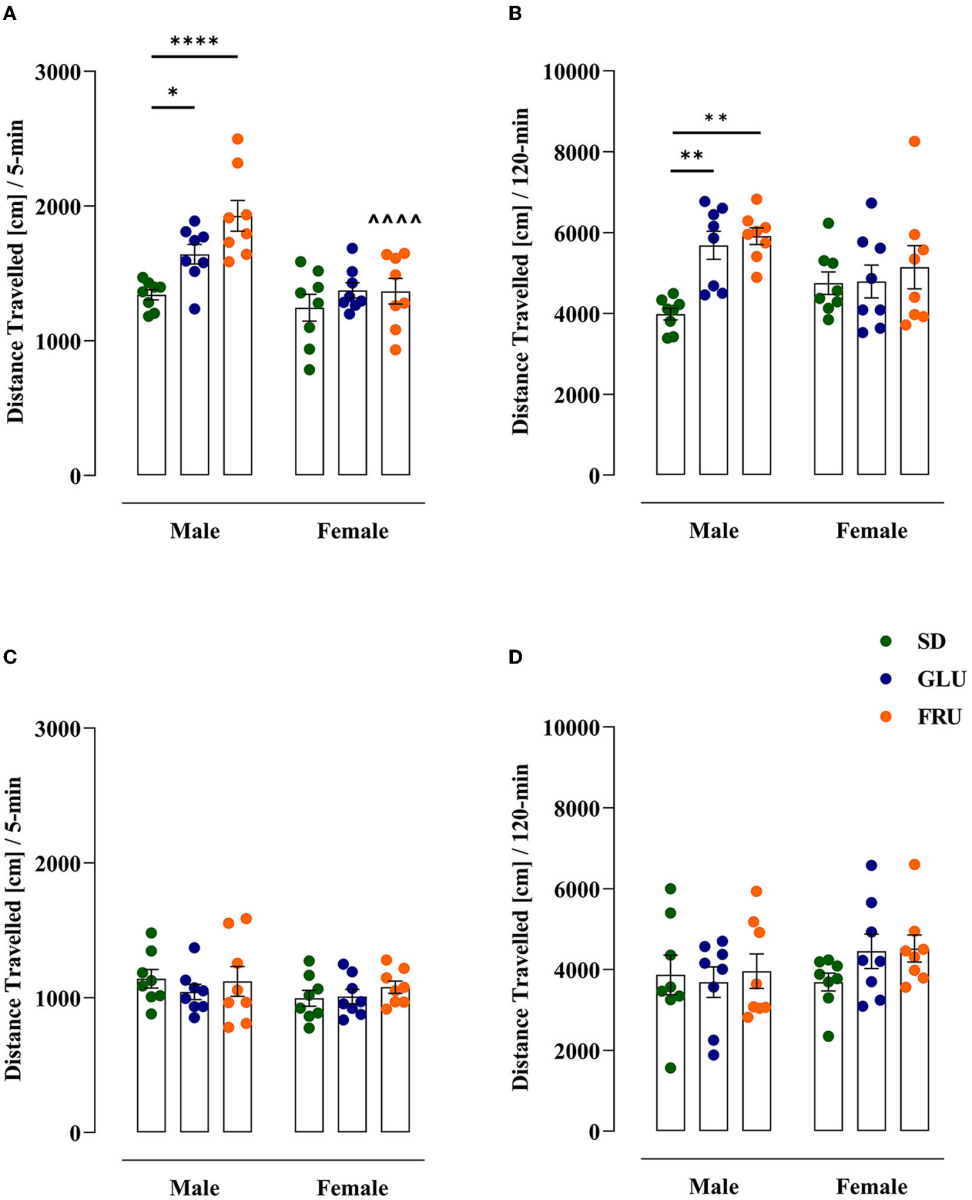
Effects of maternal glucose (GLU) or fructose (FRU) diet on adolescent (at PND27) and young adult (at PND59) offspring distance traveled. **(A, B)** Adolescent, but not young adult, GLU and FRU males showed hyperactivity during the 5-min trial. Adolescent FRU females had a reduced distance traveled compared to FRU males. **(C, D)** The 120-min trial indicated a significantly higher distance traveled in GLU and FRU adolescents but not in young adult rats. *N* = 8 rats/group. Data are comparable to the SD groups (*p < 0.05, **p < 0.01, ****p < 0.0001, two-way ANOVA or Kruskal–Wallis tests followed by Dunnett's or Dunn's multiple comparison test, respectively) or between sexes in the same group (^∧∧∧∧^*p* < 0.0001, two-way ANOVA followed by Šidák multiple comparison test).

First, we assessed spontaneous locomotor activity measured as distance traveled in adolescent (at PND 27) and young adult (at PND 59) offspring (Figure 5). Adolescent rats showed a significant main effect of diet [*F*_(2,42)_ = 9.05; *p* = 0.0005] and sex [*F*_(1,42)_ = 20.19; *p* < 0.0001], as well as an interaction effect [*F*_(2,42)_ = 3.88; *p* = 0.0283] in 5-min trial measurements (Figure 5A). Both GLU and FRU male offspring showed 22 and 44% higher locomotor activity, respectively than SD male offspring. Moreover, FRU male rats demonstrated 29% higher activity than female rats. Likewise, after a 120-min trial (Figure 5B), GLU and FRU adolescent male offspring displayed 42 and 48%, respectively, enhancement in spontaneous locomotor activity [*H*_(5,N = 48)_ = 18.57, *p* = 0.0023] compared with SD male rats. No significant interaction effects by diet and sex were observed in young adult offspring during 5- [*H*_(5,N = 48)_ = 3.57, *p* = 0.612; Figure 5C] and 120- [*F*_(2,42)_ = 0.81, *p* = 0.447; Figure 5D] min trials.

### 3.3. Effects of maternal HMD on offspring memory

**Figure 6:**
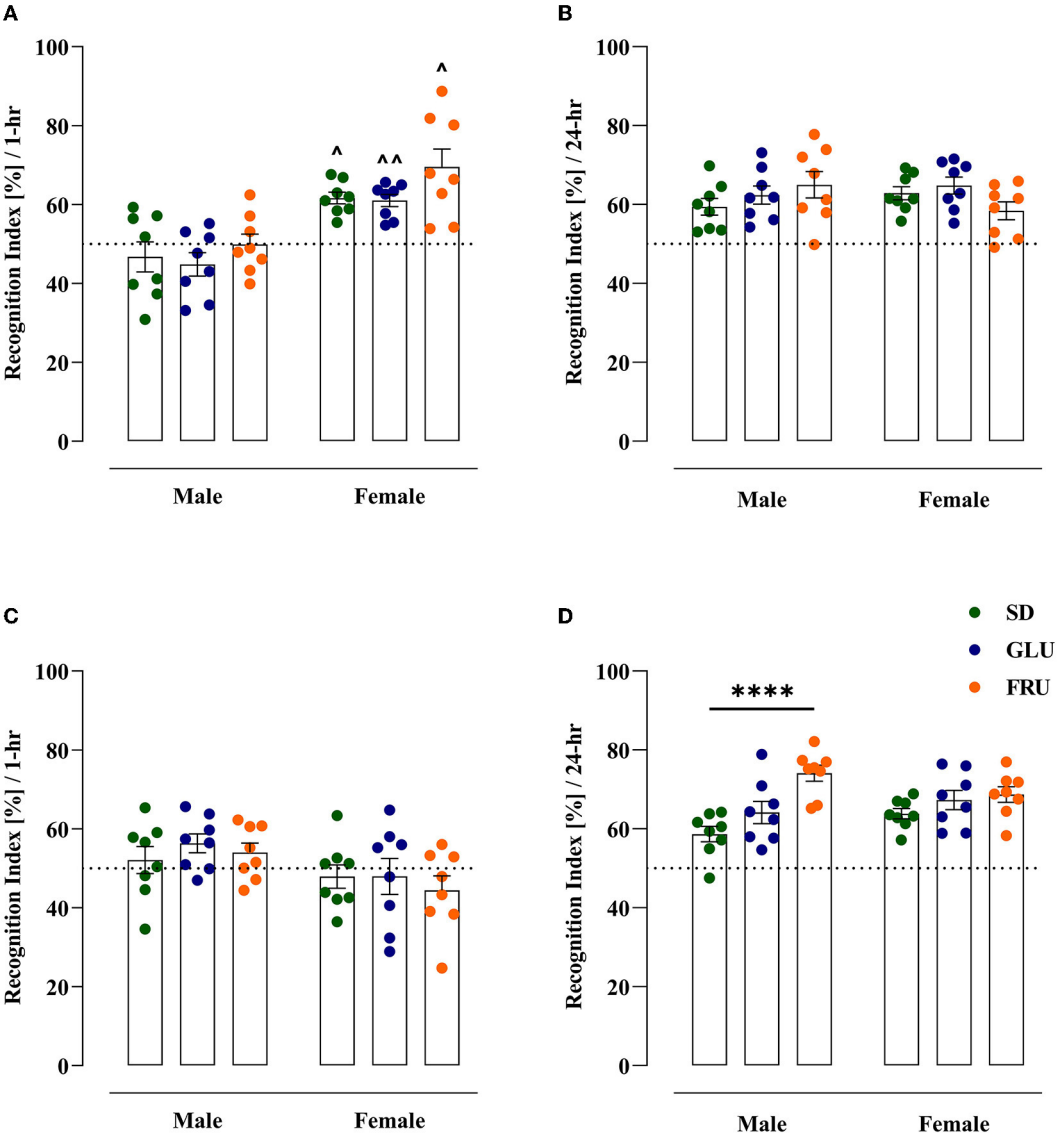
Maternal glucose (GLU) or fructose (FRU) diet changed the ability to recognize the new object in adolescent (at PND29) and young adult (at PND61) offspring. **(A)** Adolescent SD, GLU, and FRU female but not male rats showed a greater increase in the recognition index (RI) during the novel object recognition (NOR) test after a 1 h familiarization phase. FRU males and females were characterized by a higher time spent with novel objects than SD and GLU offspring. **(B)** Young adult SD, GLU, and FRU female, but not male, rats showed a decrease in RI after the 1 h familiarization phase. **(C)** After the 24-h familiarization phase, adolescent offspring showed no difference in RI. **(D)** Young adult FRU males showed a significantly increased recognition index in NOR after the 24-h familiarization phase. A larger number of offspring had an RI above 50%, which indicated a preference for the new object. *N* = 8 rats/group. Data are comparable to the SD groups (****p < 0.0001, two-way ANOVA followed by Dunnett's multiple comparison test) or between sexes in the same group (^∧^*p* < 0.05, ^∧∧^*p* < 0.01, two-way ANOVA with Welch's correction followed by Dunnett's T3 multiple comparison test).

The effect of maternal diets on short-term memory and the ability to recognize a novel object in adolescent (at PND29) and young adult (at PND61) offspring were evaluated using the NOR test (Figure 6). In adolescent offspring, SD, GLU, and FRU females displayed 24%, 26%, and 28% higher recognition indices, respectively, than male rats [*W*_(5,19.11)_ = 9.56, *p* = 0.0001] 1 h after the familiarization phase (Figure 6A). Moreover, no interaction effect was observed 24 h after the familiarization phase [*F*_(2,42)_ = 2.71, *p* = 0.078; Figure 6B].

In young adult rats 1 h after the familiarization phase, only the sex effect was detected [*F*_(1,42)_ = 7.39, *p* = 0.0095] without significant differences between male and female rats (Figure 6C). On the other hand, 24 h after the familiarization phase, significant main diet [*F*_(2,42)_ = 11.35, *p* = 0.0001] and interaction [*F*_(2,42)_ = 3.47, *p* = 0.040] effects were noticed. Interestingly, only young adult FRU male rats manifested significant (26%) increases (Figure 6D) in the recognition index in comparison to SD male rats. After the 24-h familiarization phase, both adolescent and young adult offspring had RIs above 50%, suggesting a preference for the novel object.

### 3.4. Effects of maternal HMD on offspring anxiety-like behavior

**Figure 7:**
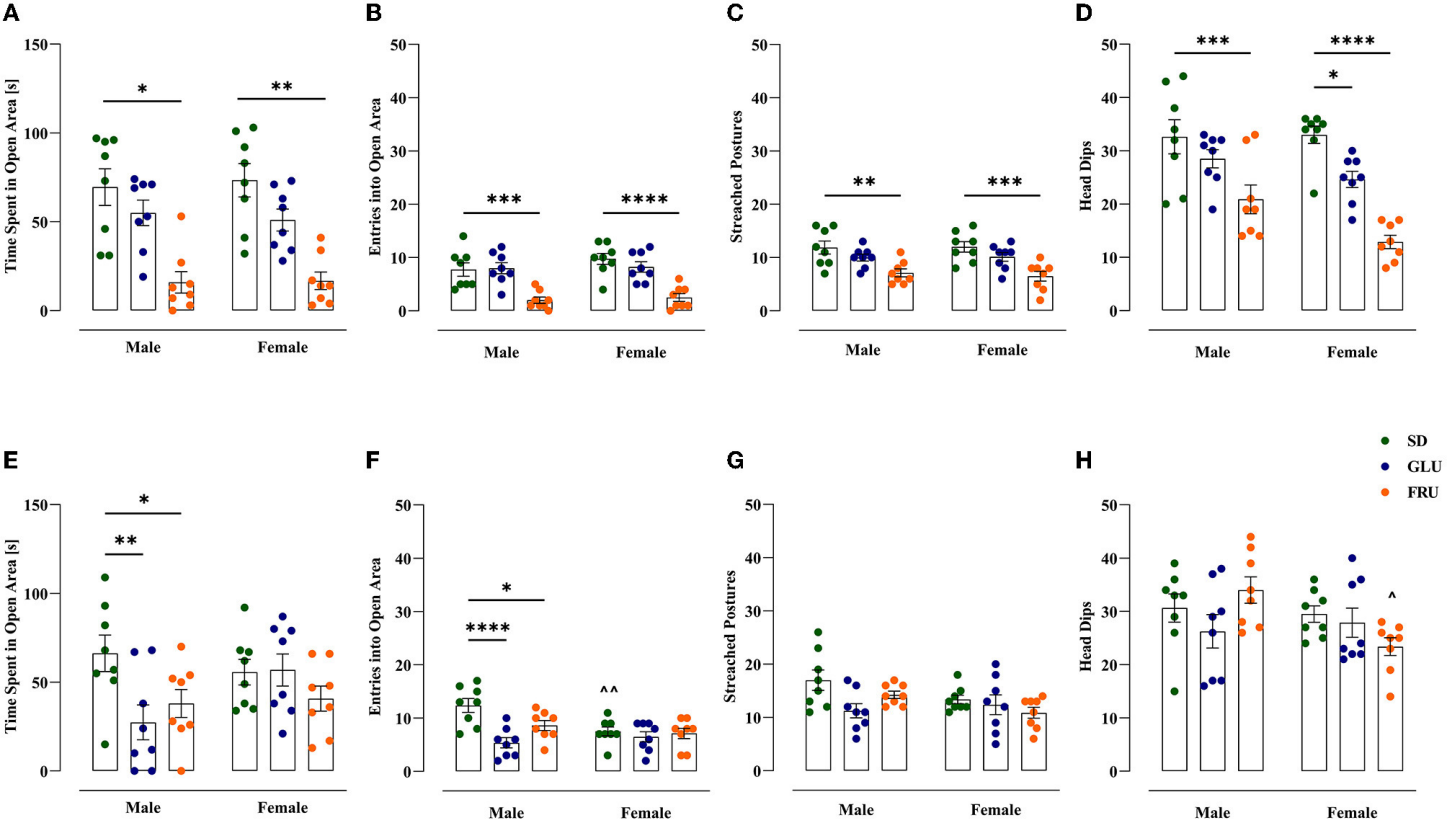
Maternal glucose (GLU) or fructose (FRU) diet increased anxiety-like behavior in adolescent (at PND31) and young adult (at PND 63) offspring. **(A–D)** Adolescent FRU males and female rats spent significantly less time in open arms of the elevated zero maze and had a reduced number of entries into the open area, the number of stretched postures, and head dips. Adolescent GLU females showed decreases in the number of head dips. **(E–H)** Young adult GLU and FRU male rats spent significantly less time in the open arms of the elevated zero maze and had a lower number of entries into the open area. *N* = 8 rats/group. Data are comparable to the SD groups (*p < 0.05, **p < 0.01, ***p < 0.001, ****p < 0.0001, two-way ANOVA or Kruskal–Wallis tests followed by Dunnett's or Dunn's multiple comparison test, respectively) or between sexes in the same group (^∧^*p* < 0.05, ^∧∧^*p* < 0.01, two-way ANOVA followed by Šidák multiple comparison test).

We assessed anxiety-like behavior in offspring using an EZM test (Figure 7). In adolescent offspring, the time spent in an open area (Figure 7A) in FRU male and female offspring was 77% lower than that in SD rats [*H*_(5,N = 48)_ = 26.25, *p* < 0.0001]. FRU male and female offspring had reduced entrance to the open area (Figure 7B) by 74% [*F*_(2,42)_ = 27.83, *p* < 0.0001] and had reduced stretched postures (Figure 7C) by 40% and 46% [*F*_(2,42)_ = 16.22, *p* < 0.0001], respectively, compared to SD offspring. In addition, FRU male, as well as FRU and GLU female offspring, showed significant (36%, 61% and 25%, respectively) decrease in head dips (Figure 7D) compared to SD [*F*_(2,42)_ = 28.63, *p* < 0.0001]. The average number of head dips in FRU female offspring was 38% lower than that in male offspring [*F*_(1,42)_ = 4.89, *p* = 0.032].

Furthermore, FRU and GLU young adult male offspring demonstrated significant decreases in time spent in the open area (Figure 7E) by 43 and 59%, respectively, compared to SD male offspring [*F*_(2,42)_ = 3.70, *p* = 0.033]. Moreover, FRU and GLU male rats entered the open area (Figure 7F) in 30 and 57%, respectively, less time than SD offspring [*F*_(2,42)_ = 7.99, *p* = 0.001]. Additionally, SD female rats showed 39% fewer entries into the open area (Figure 7F) than male rats [*F*_(1,42)_ = 4.58, *p* = 0.038]. No significance was observed in young adult stretched postures [*W*_(5,19.20)_ = 2.45; *p* = 0.070; Figure 7G]. However, FRU female offspring showed 31% lower head dips (Figure 7H) than male offspring [*F*_(2,42)_ = 3.48, *p* = 0.039].

### 3.5. Effects of maternal HMD on offspring depressive-like behavior

**Figure 8:**
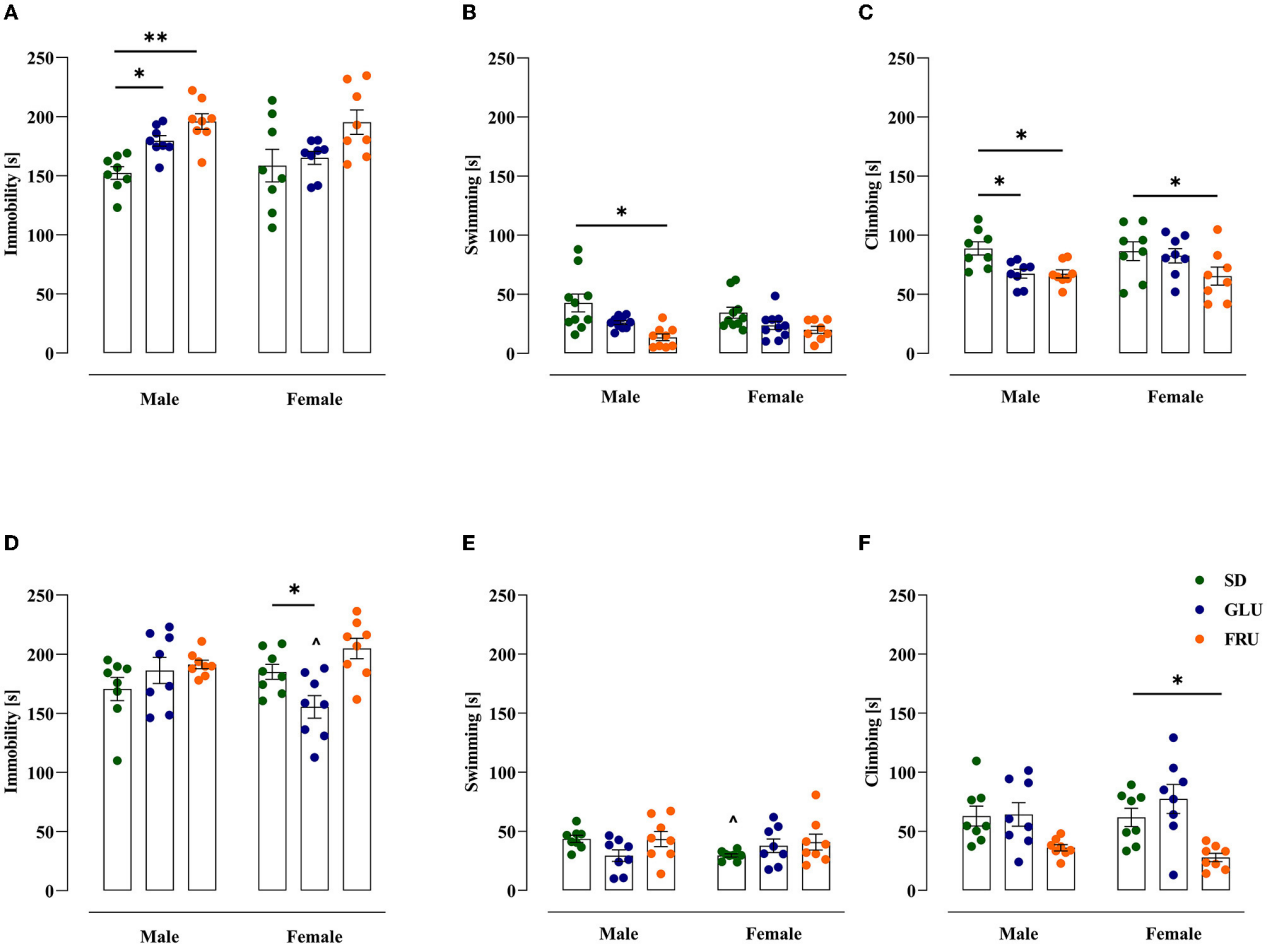
Maternal glucose (GLU) or fructose (FRU) diet display depressive-like behavior in adolescent (at PND34) and young adult (at PND66) offspring. **(A)** GLU and FRU diets changed adolescent male vulnerability to depressive-like behavior assessed as increased time spent immobile. **(B)** FRU adolescent male offspring showed decreased swimming time. **(C)** GLU and FRU adolescent male and FRU adolescent female rats reduced the time spent climbing. **(D)** Young adult GLU female rats demonstrated a reduction in time spent immobile in comparison to SD females and GLU males. **(E)** Maternal GLU and FRU diets do not change swimming time in young adult offspring. SD female offspring presented a decrease in time spent swimming compared to SD males. **(F)** Young adult FRU female offspring spent less time climbing. *N* = 8 rats/group. Data are comparable to the SD groups (*p < 0.05, **p < 0.01, two-way ANOVA with or without Welch's correction or Kruskal–Wallis tests followed by Dunnett's, Dunnett's T3 or Dunn's multiple comparison test, respectively) or between sexes in the same group (^∧^*p* < 0.05, two-way ANOVA with or without Welch's correction followed by Šidák or Dunnett's T3 multiple comparison test, respectively).

Finally, we examined offspring rat depressive-like behavior in the forced swim test (FST; Figure 8). In adolescent offspring, GLU and FRU males displayed enhanced (18 and 29%, respectively) time spent immobile [*W*_(5,19.32)_ = 6.49, *p* = 0.001] compared with SD males (Figure 8A). Additionally, compared to controls, FRU male offspring showed 24% decreased swimming behavior [*H*_(5,N = 48)_ = 16.25, *p* = 0.006; Figure 8B]. Moreover, GLU male offspring and both FRU male and female offspring demonstrated a reduction (by 24%) in climbing time [*F*_(2,42)_ = 6.41, *p* = 0.003] compared with the SD groups (Figure 8C).

In young adult offspring, significant diet [*F*_(2,42)_ = 5.48, *p* = 0.007] and interaction [*F*_(2,42)_ = 4.59, *p* = 0.015] effects were observed for immobility time. Young adult GLU female offspring showed 16% less immobility than the SD group and a 17% decrease in comparison to GLU male offspring (Figure 8D). SD female rats had a 32% reduction in swimming time compared with SD male rats [*W*_(5,18.11)_ = 4.13, *p* = 0.011; Figure 8E]. Moreover, only FRU female offspring showed reduced climbing time by 55% [*H*_(5,N = 48)_ = 21.55, *p* = 0.0006; Figure 8F] compared to SD female offspring.

## 4. Discussion

The present study showed changes in maternal monosaccharide diet intake and energy expenditure during pregnancy and lactation. For the first time, we presented significant associations between maternal FRU or GLU diet and cognitive, locomotor, and emotional dysregulation in two unrelated offspring cohorts.

### 4.1. Monosaccharide diet influences body weight and caloric intake

Our study illustrated an alteration in the dam's body weight across a high-GLU or high-FRU diet during pregnancy and lactation. Indeed, FRU female rats had significantly lower body weights than GLU and SD female rats during pregnancy. However, the FRU dam's weights oscillated with the GLU mother's weight during the lactation period. Moreover, neither of these changes correlated with the initial dam's body weight on the first day of pregnancy and lactation. Interestingly, there was no difference in total caloric intake during pregnancy, but as expected, GLU and FRU mothers received more calories from monosaccharides. In contrast to SD dams, GLU and FRU mothers consumed fewer calories during lactation, but no differences in calories from total sugar intake were observed during this period. We found that SD dams, which had a low percentage of monosaccharides in their diet (mainly from polysaccharides and disaccharides), sought to compensate for sugar calories by overeating only during lactation. These changes prove the high energy expenditure of mothers in these two different periods, where sugar is a crucial energy source for the growth and development of the fetus and later for the production of milk (42). Interestingly, GLU and FRU dam's litter sizes did not statistically differ. In turn, the postnatal weights of pups were significantly lower in GLU and FRU dams, indicating disturbances in the fetal stage of the pup's growth and development. Nevertheless, the GLU and FRU adolescent and young adult offspring's body weights during the experiment either balanced or were higher than the body weight of SD offspring. The observed sex-specific differences in body weight between diet groups were typical for any diet experiment (43).

Alternation in offspring body weight during the experiment could be due to maternal metabolic dysregulation during pregnancy and lactation or offspring fetal metabolic reprogramming by maternal GLU and FRU intake. Wistar rat dams fed 10% FRU in the water had increases in blood insulin, triglycerides, and GLU during pregnancy with a reduction in placental GLU transporter type one gene expression (44). Furthermore, FRU intake during pregnancy and lactation increased GLU transporter type five gene expression and glycogen levels with a reduction in fructokinase gene expression in the Wistar rat liver and caused sex-dependent changes in lipid metabolism in offspring (45). Moreover, Sprague–Dawley offspring rats had a higher postnatal body weight after consuming the maternal 13 or 40% water FRU solution (46). Additionally, in C57BL/6J dam mice, a 20% (w/v) FRU solution increased plasma triglycerides and cholesterol levels during pregnancy and predisposed offspring to obesity and metabolic dysfunction by postnatal high-fat diet intake (47). In the same mouse strain, pups after maternal GLU or FRU intake before and during pregnancy had less fetal body weight that was enhanced after weaning and correlated with fat mass gain compared to control offspring (48, 49). The observed sugar-dependent metabolic dysregulation was strictly associated with the different GLU and FRU biodegradation mechanisms. GLU directly enters glycolysis in each body cell, whereas FRU must be converted to glycolysis GLU byproducts in the liver as the energy source mainly for the citric acid cycle. Sugar overconsumption leads to fatty acid and *de novo* triglyceride biosynthesis, contributing to the development of overweight and obesity. GLU is mainly stored as glycogen in the liver and muscle, while much more fat is formed from FRU and stored peripherally. In contrast to GLU, FRU is the substrate of uric acid production, does not have many intracellular transporters, and is non-regulated by pancreatic hormones (50, 51).

### 4.2. Monosaccharide diet-induced hyperactivity behavior

In LMA measurements, we demonstrated that a maternal monosaccharide diet significantly disrupted spontaneous locomotor activity in offspring. For the first time, we showed that perinatal FRU and GLU intake increased the distance traveled measured during 5- and 120-min trials in male adolescents compared to male control SD rats. However, in our study, locomotor activity alterations observed in adolescent males were not manifested in another cohort of female and young adult offspring rats. Our study extends the previous observations in another research group using different rat strains. In their study, male Sprague–Dawley offspring rats (at PND50) after maternal 30% FRU-enriched water during pregnancy and lactation had higher vertical activity than females and significantly higher stereotypic behavior than control males in the 5-min open-field test (OFT) (16). Similarly, ICR mouse offspring of both sexes exposed to maternal short-term (E6–E15) dietary manipulations with FRU (30% w/v) supplementation had a specific greater increase in total distance measured in the 20-min OFT compared to the control and sucrose (30% w/v) groups (17). Nevertheless, the effect of maternal HSD on offspring locomotor activity was measured after animal habituation in the experimental cage. Moreover, the dams were fed standard rodent chow with sucrose and FRU supplementation in water. Thus, we measured acute spontaneous locomotor activity in two non-habituated age-independent cohorts at 5- and 120-min trials after maternal FRU- and GLU-enriched diets. On the other hand, it has been shown that chronic postnatal sucrose (25% w/v) or FRU (55% in diet and 10–15% w/v) consumption also increased the distance traveled in the 10-min OFT in male rats (19, 52) and male mice (20).

Hyperactive behavior in rodent studies, characterized by increased or constant movement, is considered a symptom of attention deficit hyperactivity disorder (ADHD) diagnosis in humans. In fact, in cohort and case-control trials, chronic intake of SSBs, soft drinks, and sugar binging enhanced the risk of hyperactivity behavior and ADHD symptoms in children (53). Moreover, hyperactivity and ADHD more frequently occur in boys (54, 55). The mechanism of maternal sugar action on offspring hyperactivity behavior and ADHD development is not yet well-known. Maternal malnutrition or overnutrition during pregnancy and lactation might induce neurodevelopmental effects in offspring. In mouse offspring, maternal sucrose (9 g/kg/day) consumption in pregnancy induces ADHD-like symptoms and leads to a reduction in dopamine receptors (D1, D2, and D4) and increased dopamine active transporters (17). Moreover, the higher duration of the active state has been correlated with higher expression of dopamine receptors in the offspring rat hippocampus after the maternal FRU diet (46).

### 4.3. Monosaccharide diet-prone memory impairment

In the NOR test, only the FRU diet affected recognition memory associated with short-term memory in response to novel stimuli. In comparison to the SD group, the maternal FRU diet enhanced (by 26%) the memory recognition index in young adult male rats 24 h after familiarization recognition phase II. Moreover, both young adult offspring had RIs above 50% related to a novel but not familiar object preference. Additionally, sex-specific differences were observed in recognition phase I. Female rats had a higher recognition index than male adolescent rats, but these changes were not observed in young adult offspring. In the present study, a higher recognition index observed in FRU young adult male rats could be associated with short-memory impairment or novelty-seeking behavior as a higher ability to perceive novel signals from the environment. These findings are consistent with an earlier report that a maternal HSD (with 40% sucrose) enhanced the recognition index 24 h after the familiarization phase in the two-day NOR test in young adult (at PND65) offspring of both sexes compared to control rats (36). Our results may indicate that recognition memory may not be as sensitive as spatial memory to dietary intervention. Other studies have shown that maternal HSD (with 64% sugar) decreased the preference index in the four-day NOR test in adolescent (at PND28) male Wistar rats. Moreover, a decreased novel location preference index trend was observed in adolescent and young adult (at PND70) HSD male offspring (15). In addition to HSD, a maternal high-FRU (60%) diet resulted in longer escape latency to find the hidden platform of the Morris water maze in adult female offspring, which was reversed by enriched housing (18). Furthermore, maternal FRU supplementation during pregnancy and lactation decreased latency time in FRU Sprague–Dawley offspring rats of both sexes in the passive avoidance learning test (16). A more recent preclinical study indicated that early exposure to a cafeteria diet during pregnancy and lactation impaired the performance of long-term memory tasks in adult (at PND120) male Wistar rats (56). The controversy in the findings of different maternal HSD studies may be explained by the different duration of NOR schedules, different recognition indexes measuring non-identical maternal diet composition, and offspring diversity (age, gender, and rat strain effect).

It has been well-documented that animals exposed to postnatal HSD intake had enhanced novelty-seeking behavior. Male Sprague–Dawley rats after 10% sucrose (w/v) intake for 24 days displayed an increase in exploration time with the novel object but not with a familiar object in comparison to control males (21). Moreover, 7.9% sucrose intake for 12 months significantly prolonged the time spent exploring a novel object in male Sprague–Dawley rats in the object recognition task (57). Furthermore, after 30% (w/v) FRU feeding for 9 weeks, male Swiss mice showed a higher recognition index than females and control males (22). Interestingly, female Wistar rats exposed to oral FRU (15%) supplementation for 24 weeks in a high fat-low protein diet showed increased time spent in the target quadrant and frequency of entries into the target quadrant in the Morris water maze (58). Despite the findings from these studies, several other studies have reported memory impairment after chronic, postnatal HSD consumption. Thus, postnatal sucrose consumption decreased the discrimination ratio measured in NOR (59), demonstrated poorer spatial memory in the Morris water maze (60), and decreased the amount of time exploring a novel object (61, 62) in rats.

Sugar and SSB consumption in humans influence memory and learning (63, 64). For instance, in a cross-sectional analysis of school children, higher consumption of SSB was associated with poorer performance on executive function and a high risk of executive dysfunction in children (65). Moreover, a higher intake of total sugars significantly impacted the lower prevalence of cognitive impairment scores in adult participants (66). Memory and learning processes are controlled by the hippocampus; thus, HSD can modify its neurodevelopment, neuronal plasticity, and metabolism. In a previous study, 12 weeks of maternal sucrose consumption altered episodic and spatial memory, which was correlated with a reduction in glial fibrillary acidic protein- and Nestin-expressing early-phase neurogenesis cells in the male mouse hippocampus (20). Moreover, FRU, but not GLU, consumption evoked memory impairment in rats and was associated with decreased brain-derived neurotrophic factor in the hippocampus (67). Additionally, male Wistar rats that received an FRU diet for 3 weeks showed increases in glial fibrillary acidic protein, FRU transporter (Glut-5), and tumor necrosis factor-alpha and decreases in synaptophysin, synaptotagmin, and postsynaptic density protein-95 protein levels in the frontal cortex associated with short-memory functions (68). Moreover, adolescent, but not adult, male Sprague–Dawley rats who consumed HFCS (11% w/v) for 30 days showed reduced correct hole investigation in the Barnes maze test with an increase in proinflammatory cytokine (IL-1β and IL-6) protein expression in the dorsal hippocampus (69). A recent paper also linked learning and memory impairment with the downregulation of the Wnt/β-catenin signaling pathway and changes in nervous system development gene expression in the hippocampus of offspring rats after maternal FRU intake (70).

### 4.4. Monosaccharide diet-induced anxiety-like behavior

For the first time, we demonstrated that maternal monosaccharide diet intake was sufficient to evoke anxiety-like behavior symptoms in offspring rats. As observed in our study, maternal FRU diet significantly evoked a decrease (by 77%) in time spent in the EZM with a reduced number of entries into the open area, diminished stretched postures, and head dips in adolescent offspring rats of both sexes. To be precise, the specificity of anxiety-like behavior results from the 5-min EZM test occurring in adolescent FRU offspring, especially in males, was associated with 5-min hyperactivity observed in the same rats in the LMA. Moreover, decreased time spent in the open area and reduced number of entries into the open area also manifested in unrelated young adult FRU male but not female rats. Interestingly, anxiety-like behavior appeared after the maternal GLU diet in young adult males, but it was not linked with previous GLU intake in adolescent offspring. Our results indicate that a maternal monosaccharide diet can predispose to anxiety-like disorders and/or manifest anxiety-like symptoms across the offspring's lifespan that were observed in male offspring. Similar to our findings, a few previous studies have shown that offspring exposure to a maternal FRU diet evokes anxiety-like behavior, but only when combined with saline treatment (71) or salt supplementation in the diet (72). Furthermore, maternal supplementation with 30% FRU decreased the exploratory activity of offspring of both sexes compared to control groups in the 5-min OFT (16). A recent study also showed that offspring (males and females together), after maternal 13 and 40% FRU in water intake, had higher active state duration and anxious state duration in a 5-min OFT (46).

As mentioned, our study has provided the first experimental evidence suggesting that the maternal monosaccharide diet induces anxiety-like behavior in the offspring. We also demonstrated age- and sex-dependent differences in behavioral phenotypes following the FRU or GLU diet. Our results complement recent studies that have established the relationship between postnatal sugar intake and anxiety-like behavior occurrence. Thus, male rats supplemented with 2 h of access to 10% sucrose (w/v) for 28 days demonstrated a decrease in time spent in the center area of the elevated plus maze (EPM) (23). In addition to sucrose, consumption of 30%, but not 15%, of FRU solution (w/v) for 9 weeks decreased the time spent in the open arms of the EPM in adult (at PND152) Swiss mice of both sexes (22). Likewise, chronic 18 weeks of FRU (23% w/v) feeding caused reduced entries and time spent in open arms of EPM (24), and preadolescent (at PND92) but not adult (at PND132) male rats fed a 55% high-FRU diet for 8–10 weeks reduced the time spent in the open arms of the EPM (19). Interestingly, 8 weeks of FRU supplementation in HFD (17% w/w) and water (10%) was sufficient to decrease the number of entries in OFT in male Wistar rats (73).

Notably, the maternal monosaccharide diet is sufficient to evoke anxiety-like behavior in offspring. Generalized anxiety disorders are characterized by uncontrollable anxiety, fear, and uncontrolled, exaggerated concern over endeavors. According to large population-based surveys, up to 33.7% of the population is affected by an anxiety disorder during their lifetime (74). Currently, few studies have provided evidence of a clinical or observational trial relationship between higher sugar intake and anxiety disorder events in humans (75, 76). Cohort studies have shown that sugar intake from sweet food/beverages increases the chance of incident mood disorders in men, and there is limited evidence regarding recurrent mood disorders in both sexes (31). A recent study suggested that a maternal FRU diet during gestation and lactation can affect offspring neurodevelopment. Long non-coding RNA interferes with transcript and gene expression crucial for the growth of neuronal cells in the fetal brain (46).

### 4.5. Monosaccharide diet-induced depression-like behavior

In the FST, we demonstrated that a maternal monosaccharide diet enhanced responsiveness to an acute stressor. In adolescent male rats, both GLU and FRU diets increased immobility and decreased climbing time. Only the FRU diet reduced the swimming time in male and climbing time in female rats. Moreover, in young adult offspring, a specific diet effect was observed in female rats. A GLU diet decreased immobility time, while an FRU diet decreased climbing time. The specificity of the increased depressive-like phenotype observed in adolescent males in the FST confirms the hyperactivity and anxiety-like behavior observed in the same individuals previously tested. To the best of our knowledge, there is a lack of data in the literature showing the effect of maternal HSD on the development of depressive-like behavior in offspring. Thus, our novel results establish behavioral stress-responsiveness after the maternal monosaccharide diet during offspring maturation. Several studies have demonstrated the association between postnatal HSD (mainly fructose) intake and a reduction in struggling and floating time with increasing immobile time. For instance, female Wistar rats treated with 23% FRU for 18 weeks showed increased immobility time (24). Likewise, a 55% high-FRU diet for 10 weeks evoked greater immobility time correlated with an increase in blood corticosterone measured after a 10-min FST in rats (at PND 116–120) of both sexes (77). Similarly, preadolescent (at PND94) male rats supplemented with a 55% high-FRU diet for 8–10 weeks showed an increased immobility time associated with greater corticosterone ejection and decreased struggle (19). A 30% FRU solution (w/v) consumption for 9 weeks increased immobility time in the tail suspension test in adult (at PND151) Swiss mice of both sexes (22). At the molecular level, high-FRU diet intake changed the hypothalamic transcriptome in preadolescent rats, notably corticotropin-releasing factor signaling and pro-opiomelanocortin processing (19).

Behavioral despair is a visible predictor of depression disorder development in humans. Depression is a common mental disorder accompanied by low mood and aversion to activity. Approximately 280 million people, including 5% of adults in the world, suffer from depression symptoms. Depression results from a complex interaction of social, psychological, and biological factors. Human research has revealed that high consumption of foods rich in added sugars and sweetened drinks increased the risk of depression in subsequent years (78), resulting in a high incidence and recurrence of mood disorders (79). More recent studies have confirmed that excessive consumption of sugar-sweetened soft drinks was associated with an increased risk of depression in adults and adolescents in Asia (80). In overweight individuals, increased consumption of SSBs was associated with an increased incidence of a diagnosis of depression (81). A recent cross-sectional study indicated that more than medium SSBs or fast-food consumption may lead to increased stress, depressive symptoms, and suicidal ideation in Korean adolescents (82).

## 5. Conclusion

In conclusion, our findings highlight the important role of maternal nutrition during pregnancy and lactation in proper offspring development. The results from behavioral screening using LAM, NOR, EZM, and FST tests showed that a maternal monosaccharide diet is a sufficient factor for changes in the emotional status of offspring. Herein, we demonstrated for the first time that maternal GLU- and FRU-enriched diets evoke diet-specific and sex-dependent behavioral alterations in unrelated offspring rats. Since the effects of monosaccharide consumption have not been adequately studied in humans, we suggest that the maternal metabolic dysregulation caused by overconsumption of GLU or FRU during pregnancy and lactation can both reprogram offspring metabolism and/or predispose them to behavioral disorders associated with sugar-dependent mechanisms. Based on the behavioral data, future research focusing on maternal monosaccharide diets may provide important clues to mental disorder development and prevention mechanisms.

## Data availability statement

The original contributions presented in the study are included in the article/supplementary material, further inquiries can be directed to the corresponding authors.

## Ethics statement

The animal study was reviewed and approved by 2nd Local Institutional Animal Care and Use Committee (IACUC) in Kraków.

## Author contributions

MF: conceived, designed, coordinated the study, and contributed to writing the manuscript. KWi: designed and performed the study, analyzed the data, and wrote the manuscript. KWy: performed the study and analyzed the data. AS: participated in rat generation and behavioral assays. All authors read, revised, and accepted the final version of this manuscript.
